# Draft Whole-Genome Sequence of the Alkaliphilic *Alishewanella aestuarii* Strain HH-ZS, Isolated from Historical Lime Kiln Waste-Contaminated Soil

**DOI:** 10.1128/genomeA.01447-16

**Published:** 2016-12-29

**Authors:** Zohier B. Salah, Simon P. Rout, Paul N. Humphreys

**Affiliations:** Department of Biological Sciences, School of Applied Sciences, University of Huddersfield, Queensgate, Huddersfield, United Kingdom

## Abstract

Here, we present the whole-genome sequence of an environmental Gram-negative *Alishewanella aestuarii* strain (HH-ZS), isolated from the hyperalkaline contaminated soil of a historical lime kiln in Buxton, United Kingdom.

## GENOME ANNOUNCEMENT

The genus *Alishewanella* was first described by Vogel et al. (1) following the isolation of *A. fetalis* strain CCUG 30811^T^ from the autopsy of a human fetus in Sweden. The genus has received further attention following isolations from tidal flats (2), mountainous lakes (3), landfill soils (4), industrially contaminated soils (5), and traditional fermented foods (6). This genus has received particular attention because of its bioremediation potential and broad temperature and pH ranges for growth (7–9). More recently, this genus has been observed within a floc-forming, mixed microbial community surviving at a pH of 11 (10).

The soils of a historical lime kiln waste deposition site in Buxton, United Kingdom, are hyperalkaline (pH 10.8 to 11.7) as a result of interaction with these deposits and percolating rain waters (11). A subsample of soil (5 g) was mixed with anoxic mineral medium (12) supplemented with cellulose degradation products (13) and adjusted to pH 12. Following incubation at 25°C for 30 days, a sample of the mixed culture was streaked onto fastidious anaerobe agar (pH 9.5; LabM, United Kingdom) and then incubated anaerobically (10% H_2_, 10% CO_2_, 80% N_2_; DW Scientific, United Kingdom) at 25°C for three days. A single colony was selected and further purified through subculture and was determined to be capable of growth at between pH 7 to 11 with an optimum of 9. Genomic DNA was extracted using a commercial kit (Ultraclean microbial isolation kit; Mo Bio, USA), and the bacterial species was identified as having a (99.6%) homology to *A. aestuarii* strain B11 by 16s rRNA sequencing.

A whole genome was obtained using Illumina HiSeq 2500 technology, generating paired-end 125-cycle sequence reads (BaseClear, Netherlands). Illumina CASAVA and CLC Genomics Workbench version 8.5.1 were used to generate FASTQ sequence files and assembly, respectively. Scaffolds or supercontigs were generated by linking the contigs (14). Finally, the bacterial genome was annotated via the NCBI Prokaryotic Genome Automatic Annotation Pipeline (15) and RAST server (16). In this study, the whole-genome sequence of *A. aestuarii* strain HH-ZS comprised 3,531,586 bp, encoding for 3,304 putative coding sequences, of which 71 have been classified as pseudogenes and 3,236 as hypothetical proteins; 3,165 were predicted to form known functional proteins. The genome has a G+C content of 51.0% and contains 68 genes encoding rRNA (5S, 16S, 23S), 60 tRNA, and five ncRNA. RAST system annotation assigned proteins to stress response, metal resistance (As, Cd, Co, Cr, Zn) and tolerance (Cu), metabolism of carbohydrates, and aromatic compounds associated with contaminated and high-pH environments (5, 7, 10).

### Accession number(s).

The whole-genome sequence of this project (*Alishewanella aestuarii* HH-ZS) has been deposited at DDBJ/EMBL/GenBank under the accession number LZEJ00000000.
